# “What My Son Needs Is Me. What I Need Is... Guidance”: Caregiver Perspectives About Early Autism Supports Amid Changing Attitudes and Policies

**DOI:** 10.1007/s10803-025-06850-8

**Published:** 2025-05-10

**Authors:** Katherine Pye, Lisa Gold, Ha N. D. Le, Teresa Iacono

**Affiliations:** 1 Institute of Health Transformation, School of Health and Social Development, Deakin University, Geelong, Australia; 2 School of Allied Health, Human Services and Sport, La Trobe University, Melbourne, Australia; 3 La Trobe Rural Health School, La Trobe University, Bendigo, Australia

**Keywords:** Autism, Preschool child, Family-centred care, Early intervention

## Abstract

The effectiveness of early supports for Autistic children has predominantly been measured in terms of changes in their development, such as language, cognition or adaptive behaviour. The benefits of early supports to children include, and are moderated by, effects on caregivers and families. We sought to understand perspectives of primary caregivers of children who, whether or not formally diagnosed, they believed to be Autistic, about the value of different aspects of early supports. We interviewed 19 caregivers, selected from a larger survey sample (*n* = 95) to cover a range of backgrounds and experiences accessing supports. We conducted reflexive thematic analysis to identify themes. We identified four themes through reflexive thematic analysis. Themes reflected common aspirations to optimise their children’s future wellbeing. Despite public funding for early supports, families’ other resources (especially time and mental load) were under pressure, impacting other activities such as employment and family relationships. Participants preferred supports that were adaptable to their changing needs, neuroaffirming and practical; they particularly valued authentic emotional support from therapists and sought providers they could trust. Our analysis suggests that family experiences of accessing supports might be just as important as the intended outcomes.

**Supplementary Information:**

The online version contains supplementary material available at https://doi.org/10.1007/s10803-025-06850-8.

Research data from high income countries have suggested that most children with a diagnosis of Autism access professional services to support their development (Kasilingam et al., 2019; Salomone et al., 2016; Yingling & Bell, 2020). These studies have suggested that allied health disciplines such as speech pathology and occupational therapy are the most commonly accessed supports, yet there is a wide range of professional services available; supports can differ in relation to such characteristics as ideology, methods, intensity, and the goals they target (Akins et al., 2014; McIntyre & Barton, 2010; Pye et al., 2024). The needs and preferences of families are also diverse (Battanta et al., 2024; McConachie et al., 2018). Children’s wellbeing is primarily the responsibility of their parents and other caregivers and, for children needing specific or additional supports, the caregiver role involves deciding which services to access. Caregivers with young Autistic children may find themselves at an unexpected turning point on their parenting journey, learning about Autism, their child’s needs and how to navigate the support system (Rabba et al., 2019; Sapiets et al., 2021; Smith et al., 2023, 2024; Snijder et al., 2022). Caregivers’ perspectives about early supports have been found valuable to newcomers (Moody et al., 2019) and are relevant to fellow caregivers, as well as to service providers designing or delivering supports, and policymakers allocating resources to early supports or competing programs.

Family-centred practices have been central to early childhood interventions for decades (Bailey et al., 1998, 2006; Dunst et al., 1991; Early Childhood Intervention Australia, 2016; Glass, 1999). In family-centred programs, service providers, such as allied health professionals, place caregivers at the heart of the care team, determining the nature of supports based on family needs and priorities (Johnston et al., 2016). Family-centred therapists seek to provide coordinated and comprehensive support that enables caregivers to achieve the goals that are important to them, in ways that align with family culture and values (Rodger & Keen, 2017). Understanding what caregivers find most helpful from supports is therefore essential to effective service provision.

As the key agents linking children to available services and implementing recommended strategies, caregivers are critical to the success of early supports for Autistic children. Although the effects of early supports on child outcomes have been extensively studied, (Sandbank et al., 2020), research into the potential impacts of parent and family factors on child outcomes is emerging. There is some evidence to suggest that parent factors, such as stress and maternal fatigue, can moderate or even counteract therapy effects on children (Osborne et al., 2008; Seymour et al., 2013; Shalev et al., 2020). In a study of 97 participants, Wicks et al. (2019) concluded that parent and family factors – and not characteristics of the child – predicted child outcomes from early intervention, further supporting calls to shift the focus of evaluations to consider caregivers (and families more broadly) as key to achieving optimal outcomes (Landa, 2018; Wainer et al., 2017).

In addition to their potential to benefit children, both direct and parent-mediated early supports have repeatedly been shown to benefit caregivers, such as by improving coping skills, reducing stress or depressive symptoms (Estes et al., 2019). It is also possible, however, that accessing supports could result in negative consequences in terms of caregivers’ time and the mental load of navigating the support system or learning new strategies. Yet, negative consequences of programs have rarely been directly investigated (Trembath, Varcin, Waddington, Sulek, Bent et al., 2022).

In high-income countries, early support services are often publicly funded. In Australia, the recently established National Disability Insurance Scheme (NDIS) funds a wide range of disability-related supports, including fees for services, based on individual need. The NDIS was introduced and rolled out over 2010–2020, replacing disability funding that had been deemed inadequate and inequitable (Productivity Commission, 2011). In the year ending in June 2023, NDIS payments to young children diagnosed with Autism totalled AUD$498 million (approx. USD$332 million; National Disability Insurance Agency (2023). While all services should be effective, publicly funded programs must also be equitable and sustainable, and there has been particular pressure to improve efficiency in the NDIS (Department of the Prime Minister and Cabinet, 2023).

Evaluations of early supports for Autistic children and their families have been based largely on child outcomes (such as cognitive gains) to determine relative effectiveness (Sandbank et al., 2020; Whitehouse et al., 2020) and economic efficiency (Pye et al., 2023). However, there is growing evidence that outcomes should be identified according to their value to young children and their families (Leadbitter et al., 2021; Waddington et al., 2024). Furthermore, Autistic adults have advocated strongly for neurodiversity-affirming supports in early childhood, prioritising positive self-identity and practical life skills over reduced Autism symptoms (Robertson et al., 2018) or improved academic outcomes (Gillespie-Lynch et al., 2017; Leadbitter et al., 2021). Autistic adults, service providers and parents have agreed that early supports should help adults to support children, modifying the environment (as opposed to the child) with a focus on child wellbeing (Bent et al., 2024; Waddington et al., 2023). Support for strength-based and affirming perspectives is increasingly evident in academic literature, however concerns surrounding the neurodiversity movement have included its potential to misrepresent the experiences of some Autistic individuals, especially those living with very high and complex support needs (Russell, 2020). Policymakers seeking to enable families to access supports that are both effective and efficient therefore need to know which outcomes and aspects of possible programs are seen by families as of most value.

Before the introduction of the NDIS and the widespread adoption of neuroaffirming practices, researchers explored caregiver perspectives on how therapists could be supportive (Edwards et al., 2016) and examined parents’ experiences navigating complex support systems (Galpin et al., 2018). Choosing which supports to access is a multi-faceted process, influenced by such factors as family values, prior experiences, available information, and practical constraints (Carlon et al., 2013; Edwards et al., 2018). Edwards et al. (2018) interviewed 14 parents of Autistic children in South Australia in 2012–2013 about their decision-making with regard to early supports. Their findings highlighted how parents’ decision-making evolved over time as they gained experience with the child’s needs and the support system. The authors described this as a journey from “parent to expert” in which parents initially relied on professional guidance, but over time, developed their own expertise in advocating for their child. Early on their journey, parents prioritised intervention effectiveness, particularly in terms of child outcomes, and described feeling a mix of hope and stress regarding the promise of early intervention. As they gained experience, some parents adjusted their priorities and sought more family-centred approaches or even discontinued therapies for their child (Edwards et al., 2018). These studies provide insight into parents’ perspectives regarding early supports before the NDIS, when funding and choice between services were more limited.

Currently in Australia, to support Autistic children, families typically access multiple allied health therapies such as occupational therapy and speech pathology (Pye et al., 2024). In an attempt to explore parental views of services accessed, Bent et al. (2023) interviewed parents of children who had attended an intensive university-based service implementing a naturalistic, behavioural and developmental intervention (NDBI). Parents described gratitude for the opportunity to participate in the highly regarded program and the security it offered them during a challenging time in their lives. They also reported feeling abandoned when it came to leave the program and continue their journey. However, few families in Australia have access to this type of program, instead using community-based services that incorporate eclectic approaches with varying evidence of effectiveness (Pye et al., 2024). Gaps remain in our understanding of the range of experiences among families, in particular in relation to the outcomes they prioritise and the aspects of supports they find most valuable in the current context of individualised NDIS funding and increasingly popular neuroaffirming practice.

Reported here is the qualitative follow-up of a quantitative, cross-sectional survey. Through the survey, we collected data about the services accessed by children believed to be Autistic and the health-related quality of life (HRQOL) of caregivers and family experiences (Pye et al., 2024). In brief, survey findings supported previous indications that caregivers of young Autistic children continue to experience lower HRQOL than other population groups, and their children’s Autism-related support needs are just a small driver of access to services and families’ experiences. In conducting follow-up interviews with survey participants, we sought to understand caregivers’ perspectives about the value of various aspects and outcomes of early supports.

## Methods

### Design

Caregivers from a subsample of survey participants were individually interviewed. This approach was implemented to enhance qualitative data and enrich our interpretation of quantitative findings (Collins et al., 2006). The study was approved by the Deakin University Human Research Ethics Committee (2021-446). Participants provided informed consent before commencing the survey and opted in to follow-up interviews.

### Community Involvement

The research team included a range of perspectives and experiences: professional (speech pathology), academic (speech pathology, disability and child health economics) and personal (as parents of Autistic children). Autistic individuals were not involved in the study design or interpretation beyond informal discussions within our personal and professional networks.

### Participants

Participants were eligible for the survey study if they were primary caregivers of children who (a) they believed to be Autistic (formally diagnosed or not), (b) had not yet commenced formal schooling, and (c) had lived in Australia for at least the past 12 months. The online survey was promoted via known and unknown research and support groups and social media. Further detail about the recruitment, survey and interview methods have been published (see Pye et al. (2024). From the 95 completed surveys, we purposively sampled participants likely to have ranging experiences and perspectives in relation to early therapeutic supports. We interviewed 19 caregivers with varied cultural/linguistic backgrounds, education levels, geographic locations and children’s level and type of support needs (see Table 1). We did not seek to determine a sample size a priori or in situ based on data saturation (Braun & Clarke, 2021), but taking into consideration our study aims, available resources, the number of survey participants and our strategy for interviews and analysis (Malterud et al., 2016), we anticipated that we would interview up to 20 participants, stopping after we felt we had interviewed the widest range of experiences likely from those available.

**Table 1:** Sociodemographic characteristics of participating caregivers

Characteristics	Interview sample (*n* = 19)
Child age (months; mean, range)	49.05, 34–69
Child gender (male: female)	14:5
Caregiver age (years; mean, range)	37.32, 30–54
Caregiver gender (male: female)	2:17
Child Autism diagnosis (reported)	15
Child Autism-related support needs:	
3 (Very substantial support)	3
2 (Substantial support)	14
1 (Some support)	1
Unsure	1
Reported developmental delay	14
Household income:	
Low	8
Middle	7
High	3
Not stated	1
Caregiver education:	
Low	4
Middle	5
High	10
Remoteness:	
Outer regional	2
Inner regional	2
Major cities	15
CALD identity:	
Australian	12
Other*	7
Adults at home (mean = 2.0):	
1	3
2	14
3–4	2
Children at home (mean = 2.0):	
1	4
2	14
6	1
No. therapeutic supports accessed (mean, range)	2.67, 0–4

*Included Indian, Pakistani, Filipino, Irish, Italian, New Zealand, None

### Data Collection

Individual interviews were conducted via video call (using Zoom™ or Microsoft Teams™) by the first author in September-November 2022, with phone call or camera-off options offered. Interviews of approximately 45 minutes’ duration, and were semi-structured to (a) elicit detailed service use information (Pye et al., 2024) and (b) explore participant perspectives about various aspects of early supports. A copy of the interview guide is provided in Supplementary Material 1.

Automatic transcriptions generated by the video software were edited by the first author. In most cases, word repetitions and interjections were removed to facilitate reading the transcripts, but hesitations and expressions of emotion were noted in brackets (e.g. [pauses 5 s]; [sighs]).

### Data Analysis

We undertook reflexive thematic analysis (Braun & Clarke, 2022) of interview verbatim transcripts, underpinned by a critical realist framework (Pilgrim, 2020). We held assumptions that, not only do *actual* situations and events vary across individuals, but individuals’ experiences are also *subjective*, and *reality* is a construct of both that can, in turn, be represented and interpreted variously. Therefore, we considered ourselves a subjective element within the research, as opposed to external observers seeking to identify or represent an ultimate “truth”.

The first author conducted all interviews and analysed all data. Regular discussions were held between the first and last authors throughout the coding and theming process, not in an attempt to reach consensus, but to notice and explore possible interpretations of, and relationships between, participants’ expressed views. Upon initial familiarisation with interview data, we felt that the range of perspectives expressed aligned well with the range of experiences represented in survey data after 16 interviews. We conducted three further interviews, bringing the total sample to 19 caregivers.

We were guided by Braun and Clarke’s (2022) six phases of reflexive thematic analysis, including intentional co-occurrence and revisiting of phases as part of the analytic process. Analysis began with initial familiarisation via conducting and transcribing interviews and making brief notes. The first author developed code labels and identified candidate themes. Further discussion of themes included all authors, with final refinement occurring during the reporting phase (Braun & Clarke, 2022).

A defining feature of reflexive thematic analysis is the accepted subjectivity of researchers during the analytic process. The first author’s subjectivity was influenced by several aspects of her life: she was a tertiary-educated, white female from a rural background with 20 years’ clinical experience as a speech pathologist that included working with Autistic children and their families across a wide range of settings. This experience equipped her with a deep understanding of the early intervention landscape. Through her career, this author’s views about the part speech pathology plays in supporting children and families evolved from those of an idealistic new graduate to more realistic – and at times critical – views of an experienced clinician and parent who has witnessed the shortcomings of the support system. She developed an interest in the broader context of the social support system, social determinants of health and allocation of limited resources to yield positive outcomes at a population level. One of her two children has several neurodivergent diagnoses, including Autism. She conducted interviews and analysis through this lens of her own experiences.

We approached the analysis from the bottom up, seeking to capture and organise participants’ perspectives rather than impose our own views or other existing framework on the analysis. Throughout coding and theming, we returned often to the transcripts to review the conversational context of statements we had coded. A journal was kept by the first author to facilitate the reflexive process.

## Results

**Fig. 1:**
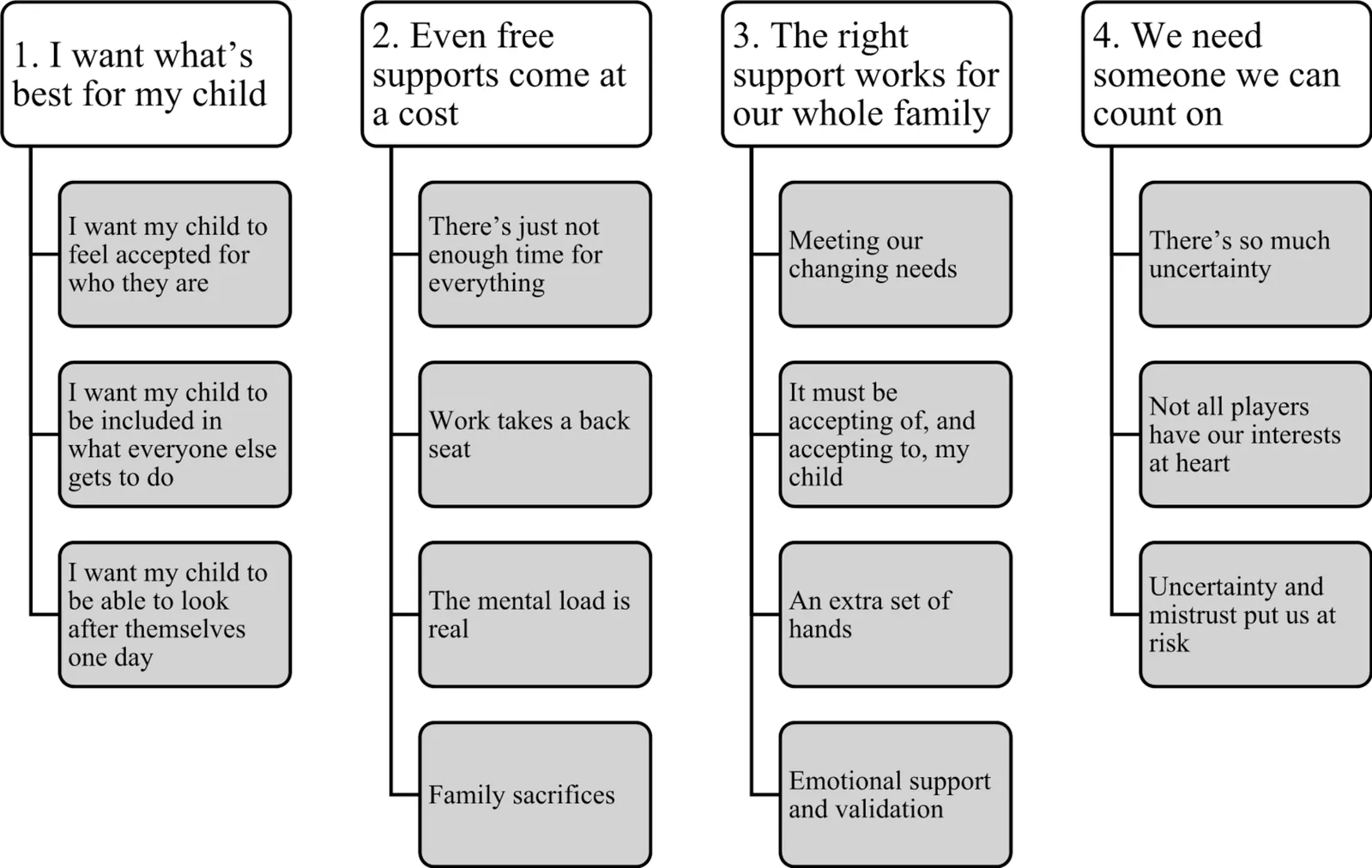
Organisation of themes and subthemes

Four themes were identified, as shown in Fig. 1. Participants are identified by number to protect their true identities (e.g., P01, P19). Although we did not directly ask participants about their own Autistic identities, five participants stated that they were diagnosed with Autism (and a sixth with ADHD). Eight participants mentioned their familiarity with Autism through work in allied health, education and/or disability.

### Theme 1: I Want What’s Best for My Child

Participants shared a strong and clear priority in what they wanted for their children. This broad aspiration was essential for them to be safe, happy individuals in the long term.

#### I Want My Child To Feel Accepted for Who They are

In particular, participants focussed on psychosocial wellbeing, driven by acceptance (by others and themselves) for who they were. These sentiments tended to be expressed with an undertone of fear that the future might not be entirely positive for an Autistic child. For Autistic parents, this reflected their own negative experiences in childhood. P1 hoped that supports in the short and longer term might protect her son from psychosocial harm that could come from societal unacceptance:

I guess longer term [I would also want] psychologies, child psychology type services to combat […] the social difficulties that he’s likely to face, and like that I faced as a child, and like early intervention to try and prevent or counteract some poor mental health outcomes, I guess.

Other participants had observed or learnt that Autistic people could be ostracised, as P9 shared, “I guess my fears would be that you know, that he just wouldn’t be included, or that he wouldn’t feel that he belonged. Sorry. […] it can become quite emotional.” Participants did not want their children to have to pretend to be someone they were not, and several parents specifically stated that they did not think academic success or reduced Autistic behaviours were important, as long as their children were happy. Participants who had observed impulsive behaviour in their children expressed fear that they could cause or come to physical harm, as expressed by P15:

…the hardest thing is not always knowing what he’s going to do. […] he’s getting taller and taller, and like I’m not always going to be able to wrap him up in Lycra. And I guess just worrying that he’s gonna do something really stupid and really hurt someone, that keeps me up at night.

#### I Want My Child To Be Included in What Everyone Else Gets To Do

Some participants simply wanted their children to access the same opportunities as their non-Autistic peers. P9, who had professional experience in specialist schools, wanted his son to avoid segregated education: “Why should a child have to be catered for at a specific location that’s not in their community, that’s not with the kids that are living next door to them?” P17 extended this sentiment beyond childhood when describing what she wanted for her daughter: “[I] just [want to] make sure that she has opportunity for everything like not very different to her [non-autistic] brother.”

#### I Want My Child To Be Able To Look After Themself One Day

Independence was another important long-term outcome desired by participants like P17, even though their children were still very young: “I think just like, independence as an adult. I know she’s only four, but you have to project into the future, really.” Being independent in the future included managing socially and emotionally, as P11 said:

When I […] feel like he can manage on his own like, control his feeling, react to the situation appropriately. At that time he don’t need me, he can do it on his own at that moment I know he don’t need any help. He can overcome problems on his own.

### Theme 2: Even Free Supports Come at a Cost

Participants were clearly committed to doing whatever they could to support their children. They were largely very grateful for the NDIS funding, some noting that they could never afford to pay privately for the supports they accessed. Despite their commitment, and the financial burden of therapist fees being borne by the insurance scheme, many families were struggling with the scarcity of other resources – in particular, time – which often meant making sacrifices that, for some, threatened the sustainability of their current situation. Some of these comments were expressed with frustration or guilt when difficult choices had to be made.

#### There’s Just Not Enough Time for Everything

Time was consistently scarce across participants, limiting families’ access to supports, employment and community. This sometimes related directly to the time they needed to spend with their children, as P08, who had two Autistic children, expressed, “The house is falling apart man, because we never get to do stuff. The kids need someone there all the time.” Additionally, time participants spent navigating the support system and attending children’s therapy sessions was often considerable.

#### Work Takes a Back Seat

Some participants mentioned that they worked less, or not at all, because of the time needed to access their child’s supports. This experience was captured by P16, who said, “It’s too hard to work. Because Mondays are speech, Tuesday’s my only free day, I still drop them off [at childcare], Wednesday’s OT, Thursday’s OT, and what employer’s going to let you take this much time off?”

#### The Mental Load is Real

Caregivers described feeling overwhelmed and exhausted by the mental load of navigating the system and coordinating their family’s needs. There was a sense that their mental capacity was, in a way, another resource that was being overstretched. As P06 said, “It can be really exhausting and talking to all the different therapists and having to keep track of them.” Even participants with professional experience of the system found is difficult to navigate and access supports. As P03 said,

I find it very challenging, and […] I work in the system. So I can’t imagine how challenging it is for some families, accessing different therapists, trying to find the right one, trying to find the ones that fit, getting into the NDIS in the first place, jumping through all the hoops, knowing what you can access and what you can’t. It’s near impossible to be honest, to get everything that you need.

She went on to point out that caregivers who were neurodivergent themselves would be particularly impacted by this issue:

…trying to access these supports with ADHD or being Autistic, makes it ten times harder because it’s not designed for a neurodivergent brain at *all*. Like all the paperwork and phone calls and finding the people and research. It’s a lot.

In addition to navigating the system to access and coordinate supports, the heavy mental load was also attributed to implementing therapy strategies at home. P05 described this in the following:

So if you’ve got a child, that […] doesn’t really want to do anything that you’ve been learning, […] like change is his big thing. He does not like change. But any sort of change that we have in our environment, I have to do it really slowly and make him sort of unaware that it’s being changed. So that I think’s been more challenging for the burden on me.

Participants noted that available supports were largely focussed on their children’s direct needs, without addressing their own mental health. P19 stated:

There is a lot of support for kids around, in terms of therapies and as well, there should be an awareness thing for parents as well, that you know, when you take care of kids you go through emotional breakdowns, you go through emotions.

#### Family Sacrifices

Participants described various difficult choices or activities they had foregone in order to access supports for their Autistic child. P17 had to decide which of her children could access speech pathology because their therapist’s availability was limited: “During the pandemic we had to choose which [of our children] to do speech therapy. […] So that was hard.” Participants had often sacrificed time for themselves to unwind or invest in their own health – as captured by P16:

I’m very hard on myself, I’m very down on myself, I’m very neglected with my health. Finally going to the dentist tomorrow afternoon after 3 years, I’ve gotta get new glasses, I’ve got to make an appointment. Everything’s just on top of me at the moment. Next week I’ve gotta go into the hospital…we have to wait and see what happens, maybe I need a little surgery after that, and I’m like “Oh no not now, the kids are so little, you know, I just don’t want any news – well bad news especially”. I wanna get them off to school next year, I wanna get them into a routine, I wanna finish off this year, and it’s just… yeah.

Some participants acknowledged that compromising their own health was not in their child’s best interests, as P03 articulated:

I *am* her co-regulation. If I’m not able to have the time and space to meet my own needs and look after the rest of the family, I’m not able to support her, which puts her in a much more disabling place that what she was before.

P04 described the impact of her own limited resources on the relationship between parents:

We separated because we couldn’t agree on parenting - and many other issues, but a lot to do with [my child’s] diagnosis […]. I didn’t eat, sleep, you know, like It’s… parenting a special needs child is just next level. So I didn’t have any goals apart from staying alive. So yeah, I think that’s a very important conversation.

Caregivers with more than one child expressed concerns about spending more time with their Autistic child than siblings. While related to the scarcity of time – and perhaps mental load - to do everything (subthemes 2.1, 2.3), these competing family priorities seemed to evoke particular guilt or inner conflict, as P09 expressed:

I think what’s been really… a balancing act, has been how we afford [our non-autistic child] the same sort of attention. It’s just, you know, because of the nature of the supports that we give for [our Autistic child], […] it does feel like at times we’re spending more time with one than the other.

P16 took this to heart: “I really feel sorry for [my other child], he gets much less attention. [My Autistic child] is just… it’s just really hard [sighs].”

### Theme 3: The Right Support Works for Our Whole Family

Although the overarching priority for families in terms of long-term outcomes was clear (Theme 1), caregivers seemed less sure about the medium-term outcomes they hoped to achieve with early therapeutic supports. Families accepted that they were playing a long game, rather than articulate specific objectives they wanted to achieve during their child’s early years, they described what truly helpful supports would look – or feel – like, in the here and now.

#### Meeting Our Changing Needs

Another feature of most valued supports was that therapists adapted to families’ changing needs. Examples included needing more or less support at different times because of family situations or children’s development. P14 had a helpful arrangement to address this fluctuating need for support:

We took a break for a little while, because he had calmed down. But at the start of the year when he transitioned to [pre]school […] he became more and more heightened, and we’ve gone back. So […] if we cease sessions with her, we’re still on her books, and we just need to let her know if we want to come back.

P05 described her own difficulty adjusting to her son’s changing needs as he developed:

Obviously kids on the spectrum, its fluid, like […] They’re going to have their days when they’re off, or when they’re not sort of, you know, so I guess that’s been the hardest thing […]. You know, you’re doing really well, and then you get those setbacks or change in routine.

Several participants expressed a preference for supports *not* to be too goal oriented. They noted that setting, and holding to, specific goals for their child could limit the flexibility of supports.

#### It Has to be Accepting of, and Acceptable to, My Child

For some participants, it was essential that supports were acceptable to their child. Indications of this included children enjoying therapy sessions and experiencing positive rapport with professionals:

[Our speech pathologist] gives me a positive outlook on things, and […] rather than me thinking of things as a problem or an issue, she’ll turn it around to what can we do to help, and I think, having developed that relationship with her, and seeing the relationship that [my child has] developed with her, because he loves her too, he gets so excited, he’s even got a doll that he named after her. [laughs] Yeah, it’s really cute. He just loves her. So I think that’s really helped me sort of accept it a bit more. Not that I didn’t accept it, but just be more confident in my abilities I guess as a parent to help him as well. (P07)

The attitudes of professionals and ideology behind approaches they implemented were also perceived indicators of acceptance of neurodiversity. These participants provided examples of practices they considered neurodiversity-affirming:

The language [our therapist] uses is very positive, very affirming. […] No “red flag” words or anything like that. No social skills, no, behaviour therapy, none of that kind of stuff popping up, which is great, just really positive worded. (P06)

We just felt like [a previous therapist’s approach] was an attempt to sort of normalize [our child], and not really appreciating his neurodiversity. We want to surround [him] with people who value that. (P09)

People always assume that being Autistic means that a meltdown is inevitable and frequent meltdowns are normal. And in reality it’s actually really not. It’s an Autistic person in distress, and if we can meet her needs, then we’re naturally going to reduce [her] distress. (P03)

#### An Extra Set of Hands

For some, practical support with daily routines, such as that provided by support workers, offered more value than therapeutic supports aimed directly at building their children’s capacity. As P08 put it:

I know, these like therapy and things… I don’t know how helpful they are sometimes. Honestly, I just want someone to help me to be a mum. That’s what I want. And having people tell me what I’m supposed to be doing all the time, it’s not helpful.

Participants described needing practical support in their daily lives. Day-to-day demands could be unpredictable and/or need urgent attention, meaning that, due to limited resources (Theme 2), they could trump families’ long-term plans. Practical supports, when available, enabled families to access the community together, as P08 illustrated:

The support workers […] actually help us to do the things that we want. So, we’re going to the air show. […] We booked the tickets months ago, […] and we know we’ve got a support worker for each of the kids, so if I get distracted I’m not going to turn around and have [a child] go missing in a sea of people. In the middle of an airbase [laughs]. And so I know that you can actually go and relax if the kids need to go home, you know. If one kid needs to go home they can just go. And so that is what has changed everything for us.

Practical help meant caregivers could be more focussed on building relationships with their children, as for P16: “Oh, an extra pair of hands [would be] good. Someone to maybe cover what I’m doing, so I can do a bit more with them I suppose.”

#### Emotional Support and Validation

The value of emotional support was evident in the gratitude participants expressed for professionals whom they felt had been sincere in offering to help them. P19 was immensely grateful, not only for the practical help offered by her occupational therapist, but also the genuine nature of the offer:

My husband [was away]. And when I told [our occupational therapist] that I’m alone with both kids, she said, “I’m not joking, if you need me to take care of your kids, I can leave my sessions, and I can come and […] they can spend time at my house.” I mean it was a big thing and I know […] she was not just saying it. She meant it. (P19)

It seemed that service providers’ sincerity acted to share the emotional load that participants had experienced. Authenticity was appreciated by participants who sensed that their service provider was personally invested in their child. P14 tried to capture why he so valued his child’s speech pathologist, saying, “She’s really just driven to see the difference for us and for him, and she’s so proud of him when he does things.”

Therapists who related to caregivers’ experiences, including neurodivergence, were greatly valued. P08 stated:

This woman, she’s so good. She’s also Autistic with ADHD, and she’s very similar to me. So she understands how to ask me questions. She understands how to explain things. […] She’s like, “OK [P08], you don’t quite look like you’re with me.” So she’ll rephrase something, or [use] a different kind of analogy. […] I think the communication between us is just so, like I feel so comfortable.

Conversely, P08 expressed frustration when other professionals had failed in this respect:

My kids are awesome. I love my kids. […] But not validating the things that I say, the things that we do, the challenges that we have, you know, telling [me], “You’ve just got to have more […] visual resources.” Yeah, if we put another fucking visual schedule in this house, I swear my eyes will just go on strike. They’ll just pop out of my head and run away. Like, there’s so many visual things in this house that if you put another one in, you won’t see any of it. It’s just a sea of colour. [laughs] So it’s, “You just need this. […] Oh, that’s normal, kids will do [that]”. Things that just, validate our experience. Yeah, that’s what I would ask.

### Theme 4: We Need Someone We Can Count on

This final theme was evident both among participants who were enormously grateful for the dependability and genuine investment of their therapists, and among participants who felt vulnerable or let down. For some experienced participants, the value they placed on reliability came not from their own vulnerability, but from working professionally in the system, or having multiple children with support needs and knowing that they just didn’t have time to waste.

#### There’s So Much Uncertainty

Caregivers expressed great uncertainty in several aspects of their current situation, such as not knowing what their child’s future might hold. This evoked anxiety in P09:

There’s no path as to what [having a child on the spectrum] looks like. […] They all have different profiles. So it was, “Well, what is that going to mean for [my child] when he’s in school, adult…?” and your anxiety is just through the roof, because you just don’t know what that’s going to look like.

For P13, uncertainty about their child’s future made it difficult to know which supports they should seek:

I don’t know what [my child] is going to need, like in 2 years’ time […] Like one of my friends mentioned to me the other day about a psych. And I’m like, “Why? Why would I need a psych? Do I need a psych? Do I need to put my name on a waitlist now for a psych, cause I might need a psych when he starts school?”

Uncertainty extended beyond the nature of children’s current and future needs, to the availability of supports, even among families accessing regular therapy. P14 shrugged and said, “Now we’re coming to the end of the year, although the speechies will continue, I don’t know what’s going to happen to the OT.”

Another major source of uncertainty was regarding the NDIS. P10 described contradicting information about how their funding could be utilised:

But if I take on this [therapist], does that negate my ability to access this? […] What am I eligible for? That was a nightmare, and I am well versed in this stuff. Absolute nightmare. I don’t know how other people even do it.

#### Not All Players Have Our Interests at Heart

Some participants had come across bad actors in the system. Some of this perception related to the social system being unfair, with P13 expressing that the NDIS was “so corrupt and rigged”. Some reports were about paying for services with public money (i.e., the NDIS) resulting in providers being inefficient or even dishonest:

Everyone wants to know exactly how much money you have in your [NDIS] plan, and they want to spend every single cent in your funding. […] My last speech pathologist had no clue about children whatsoever, and just wanted to use all of the funding on writing reports, which meant absolutely nothing to [my child], and he doesn’t get any good from. (P13)

In some cases, it was not the process itself that caregivers felt was unfair, but providers’ over-zealous application of the rules or lack of family-centred common sense. P08 had an agent who arranged for therapists to be paid from her children’s NDIS funds, but when a payment was outstanding, she recalled that the therapist arrived and said, “Well, there’s an outstanding invoice, I think, for one of the kids. […] So I’m only seeing [the other child] today.” This was impractical when both children were home and expecting their usual sessions. Additionally, some administrative processes were not considered valuable to families, as P08 went on to capture: “I also don’t know why I’m paying for fifteen minutes of note taking when you never see any of the notes, the notes are never helpful.”

#### Uncertainty and Mistrust Put Us at Risk

Uncertainty about the system led participants to feel that they weren’t in control of the supports they could access, which linked to feeling particularly vulnerable. A sense that access to support was out of their hands was expressed by P02:

I think we’re lucky. We just got a really good speechie. Yeah, we’re very lucky, I think. So I stumbled across her through a friend. […] But yeah, I don’t know how other people go finding people.

P18 was apprehensive about advocating for more support in their child’s NDIS plan, recalling they had thought, “You know what, let’s not challenge it because we could lose everything. So we’ll just accept what they’ve given us.”

Outcomes of such uncertainty included missing out on, or paying privately for, supports eligible for NDIS funding, as was expressed by participant P85, who didn’t know they could access funding without an Autism diagnosis, and spent months (and private funds) seeking assessment before they started accessing any early supports. Some caregivers highlighted how helpful prior knowledge of Autism or the support system had been, expressing concern for families who were less well-prepared to navigate the system.

P11 had not yet accessed any supports for her child, and yearned for guidance. She linked this to her isolation as a recent immigrant to Australia and her cultural community’s lack of understanding about Autism. The stigma she experienced had been a barrier to accessing informal support from family and friends and professional capacity-building support.

## Discussion

We undertook this study to understand what caregivers with children, who they believed to be Autistic, valued or desired most from early supports. This research was driven by a need to identify, from families’ perspectives, which outcomes should be measured to meaningfully evaluate the effects of supports. Although consistent in wanting their children to be accepted for who they are in the long term, participants were less concerned with specific outcomes from early supports, focusing instead on the experience of accessing services and managing their families’ needs. Participants were, on the whole, more concerned with immediate outcomes that would enable the whole family to adapt and get on with their lives, than about teaching their Autistic child specific skills. This family-wide context had apparently been understood by some therapists, but the current system did not adequately facilitate practical or identity-affirming support. The vulnerability experienced by some families related to the early stage of their Autism parenting journeys, overlaid by the complexity and lack of trust in the support system. Overall, while focussed on their children’s futures, during their early childhood, caregivers most valued supports that were authentic, whole family centred and practical.

Our findings share similarities with previous studies of parental perceptions and priorities in relation to early supports for Autistic children. Edwards et al. (2017) found that parents interviewed before the NDIS experienced hope when they first encountered early supports, as well as a sense of urgency that could result in rushed decisions about the services they accessed. Their findings were echoed by the vulnerability expressed by participants in the current study, and perhaps the frustration over systemic issues and delays accessing early supports. Our findings are also consistent with recent research demonstrating that parents, like Autistic adults and professionals, preferred early support goals that were considered neuroaffirming and helped adults to support Autistic children (Waddington et al., 2023). The current study provides further support for calls to increase attention to practical supports (namely, disability support workers to help with daily routines) and family outcomes (Gavidia-Payne et al., 2024; Wainer et al., 2017).

Participants in this study came from a range of backgrounds, lived across Australia and accessed various types of support. One family had not accessed any formal supports, and only one other accessed an intensive, behavioural program. With this in mind, we expected the views expressed by our sample to differ from those reported by Bent et al. (2023), whose children had all participated in a university-run, intensive program. In contrast to Bent et al.’s findings that caregivers felt grateful, secure and wedded to the specific approach implemented with their children, our themes reflected strong preferences for neuroaffirming practices (including rejection of behavioural methods), and fluctuating and varied priorities in terms of therapy targets and service delivery. Although families in the intensive program may seem to have been better off than those in the current study, our findings clearly suggest that an intensive behavioural program would not suit all families.

### Implications

Neuroaffirming practice is gaining popularity in Australia and internationally. Professionals may need to learn from advocates including Autistic adults to better understand what this involves and adapt their practice to better meet the needs and preferences of their clients (Leadbitter et al., 2021); at the same time, clinical practice should be driven by a robust evidence base (Bottema-Beutel et al., 2023). The notion of family-centred care is already well-established and has been endorsed in practice guidelines (Early Childhood Intervention Australia, 2016; Trembath, Varcin, Waddington, Sulek, Pillar et al., 20222022), yet our participants described difficulties fitting early supports into their busy family lives, some even sharing that accessing supports increased their total load, rather than reducing it. These experiences prompt consideration of the optimal number of services for each family, how they might best be coordinated, and their adequacy in addressing each family’s values and needs. As societal attitudes become more neuroaffirming and neurodivergence is met with less stigma, pressure on some families to engage in early interventions may arguably become less urgent, potentially reducing the number or intensity of services accessed. This possibility aligns with previously cited findings that, as parents came to accept their child’s Autism diagnosis and understand their needs, some chose to reduce the amount of early professional support they accessed (Edwards et al., 2018). As society accepts and embraces neurodiversity, the intended outcomes of early supports may shift further away from child-centred targets to prioritise the practical needs of families, as is consistent with family-centred care (Dempsey & Keen, 2008; Johnston et al., 2016).

The perception that accessing good supports comes down to chance points to major inconsistencies in available services. While a single approach or service delivery model is unlikely to suit all families, caregivers require certainty about what to expect from providers. Without this, the NDIS’s foundation on participant-centred care is compromised, with potentially significant inefficiencies caused when families ‘shop around’ to find supports that meet their needs. In underserved areas and for families poorly equipped to navigate the support system, these inconsistencies contribute to inequities in the NDIS and related services.

### Strengths, Limitations and Future Directions

This study had several strengths. First, selecting participants from a larger sample meant that we could, to a certain extent, curate a rich set of participant perspectives. Second, interviews were conducted by a researcher with considerable professional and personal experience of young Autistic children. This experience provided a shared understanding of context, facilitating authentic interviews and sensitivity to nuances in participants’ comments. Third, the inductive analytic approach enabled us to respond to caregivers’ focus on the *experience* of accessing supports, rather than specific *outcomes* from early supports. As a result, this study has highlighted the multidimensionality of families’ experiences, in that they extended far beyond children’s developmental outcomes.

The study also had limitations. We are confident that many family experiences were not captured in our data. Most importantly, the most underserved populations, such as those with insufficient English language or literacy to participate, those living very remotely and with highly complex social and support needs, were either not captured by our survey or unable to commit to interviews. Our study design was not intended to represent all families in the broader population, warranting further research into what the most vulnerable families value most from early supports, especially as their priorities and preferences may differ from those whose needs are broadly better served. The subjectivity that is inherent in reflexive thematic analysis is also acknowledged: different authors would identify different themes from those presented here. Unlike our approach to understanding the perspectives of purposively sampled participants, quantitative or content analysis or representative samples would provide an opportunity to understand the proportions of specific preferences in the broader population, and possibly prioritisation of the issues raised. A further limitation was that we spoke to each participant once only. Follow-up conversations after analysis of initial interviews would have enabled a deeper understanding of caregivers’ views and provided the opportunity for members to review themes.

Future research is needed to further enhance current understanding of the feasibility, acceptability and effectiveness of early Autism supports from the perspectives of the most important stakeholders. Investigating the experiences of children (while accessing supports) and Autistic adults (with a history of accessing supports) is critical to considering how services can engage children and align with their needs and preferences. Evaluations that identify and measure outcomes valued by caregivers and Autistic children/adults will authentically reflect the preferences of the populations most directly impacted. Investigation into different stakeholder perceptions of neurodiversity-affirming practices and their consequences would deepen understanding of these concepts that have recently become very widely used.

## Conclusion

This study has highlighted that primary caregivers particularly value supports that genuinely validate their individual experiences, alleviate pressure on families in practical and validating ways, and respond flexibly to the changing needs of children and families. Some caregivers actively sought out neurodiversity-affirming supports, and many sacrificed other priorities in order to invest scarce time and mental load in pursuit of their child’s long-term psychosocial wellbeing. These insights have direct implications for future development and evaluation of early supports for neurodivergent children and their families.

## Electronic supplementary material

Below is the link to the electronic supplementary material.
