# Supplementary material for: “What My Son Needs Is Me. What I Need Is... Guidance”: Caregiver Perspectives About Early Autism Supports Amid Changing Attitudes and Policies
Source: J Autism Dev Disord. 2025 May 10;56(10):4121–34. doi: 10.1007/s10803-025-06850-8 (PMC13538209; doi:10.1007/s10803-025-06850-8)
Supplement: Supplementary file 1 [file 10803_2025_6850_MOESM1_ESM.docx]

SEMI-STRUCTURED INTERVIEW GUIDE

|  | **INTRODUCTION** |
| --- | --- |
|  | Greeting, thank you for time |
|  | I will tell you more about my own background at the end, but if it’s OK we’ll just get started. |
|  | Confirm details re child/fam (from survey data):   - Child name, age - Location - Diagnosis - Place in family - Childcare - Supports currently accessed - CALD background |

|  | **SERVICE USE DETAILS** |
| --- | --- |
|  | I’d like to know more about the supports you access right now. *[for each, ask these details:]*   - Discipline (including professional or AHA?) - Setting (home, clinic, childcare?) - Mode (face to face, telehealth?) - Group/1:1 - Agent (therapist, parent, hybrid, other?) - Approach implemented? - Session duration and frequency - How are you involved in the sessions? *[e.g.live coaching, watch and learn, practice/feedback; expectation of home practice or some kind?]* - Have you been offered resources for yourself (e.g. workshops, support groups, counselling)? - Why have you chosen this support? *[e.g. close proximity, friend recommendation, first available, specific program]* - How are you paying for this support? *[eg NDIS, Medicare, private health insurance]* - Do you know the fees they charge (eg per session/hour)?   And how do you feel about this particular support? Do you feel well-supported? *[Follow participant’s lead here to understand their experiences]* |
|  | Is there one support that you’re finding most valuable? Why? *[explore outcomes]* |
|  |  |
|  | Are you waiting for other supports? |
|  | Have you started any supports but not continued?   - Why did you stop? |
|  | Are there supports you would like to access, but haven’t been able to?   - Why not? *[i.e., what are the barriers: eg, lack of availability, parent time, waitlists]* |

|  | **OUTCOMES** |
| --- | --- |
|  | What do you hope to get out of supports you access? |
|  | Why are those things important to you? |
|  |  |
|  | Are there goals or outcomes that you feel aren’t being addressed by your supports, that are important to you? |
|  |  |
|  | Do any of these outcomes stand out as being the most important to you? |
|  |  |
|  | FAMILY-BASED OUTCOMES:  Are any of these goals important [if not discussed already]?   - Help parents know how to parent/teach their child: boundaries, expectations, independence, relationships, skills - Reduce parent stress in relation to interactions with your child/ren - Increase parent sense of competence - Help family go out in the community more (eg parks, festivals, restaurants, birthday parties) - Help parents understand autism and their child - Help family access relevant supports, funding etc - Help parents play with their child - Improve family QOL - Teach others about autism/your child   CHILD-CENTRED OUTCOMES:   - Reduce autism symptoms - Increase child IQ, academic success - Increase child’s ability to cope in different environments - Help child develop friendships - Safety |
|  | How would you know a support was working well for you? |

|  | **EARLY DAYS EXPERIENCE** |
| --- | --- |
|  | What has helped you most during the early stages of your child’s autism journey? |
|  | What has made the process more difficult? |
